# DJ-1 in Parkinson’s Disease: Clinical Insights and Therapeutic Perspectives

**DOI:** 10.3390/jcm8091377

**Published:** 2019-09-03

**Authors:** Mariaelena Repici, Flaviano Giorgini

**Affiliations:** 1 School of Life and Health Sciences, Aston University, Aston Triangle, Birmingham B4 7ET, UK; 2 Department of Genetics and Genome Biology, University of Leicester, Leicester LE1 7RH, UK

**Keywords:** DJ-1, Parkinson’s disease, biomarker, neuroprotection

## Abstract

Mutations in the protein DJ-1 cause autosomal recessive forms of Parkinson’s disease (PD) and oxidized DJ-1 is found in the brains of idiopathic PD individuals. While several functions have been ascribed to DJ-1 (most notably protection from oxidative stress), its contribution to PD pathogenesis is not yet clear. Here we provide an overview of the clinical research to date on DJ-1 and the current state of knowledge regarding DJ-1 characterization in the human brain. The relevance of DJ-1 as a PD biomarker is also discussed, as are studies exploring DJ-1 as a possible therapeutic target for PD and neurodegeneration.

## 1. Introduction

DJ-1 is a small, highly conserved protein of 189 amino acids, which is ubiquitously expressed and dimeric under physiological conditions. In humans, DJ-1 is encoded by the *PARK7* gene, which was first linked to early onset, familial forms of Parkinson’s disease (PD) in 2003 [1]. Bonifati and colleagues found that loss of DJ-1 function—either due to a deletion of 14,082 bp in a Dutch family or a homozygous point mutation (L166P) in an Italian family—caused disease with parkinsonian features. Clinically, PD patients with DJ-1 mutations exhibit an early onset of dyskinesia, rigidity, and tremors, followed by later manifestation of psychiatric symptoms, such as psychotic disturbance, anxiety, and cognitive decline, and generally respond well to L-DOPA treatment [1,2,3]. Since this initial study, a great number of mutations in DJ-1 have been identified, with some more clearly linked to pathogenesis than others (see Table 1).

Several studies have deciphered the consequences of different DJ-1 mutations on its three-dimensional protein structure [18,19,20,21]. This work finds that while some mutations completely disrupt dimer formation, others do not greatly perturb this, suggesting alternative reasons for DJ-1 mediated pathogenesis. In contrast, very little is known regarding the neuropathology in brains from patients carrying DJ-1 mutations. Indeed, only one brain from a patient with DJ-1 linked PD (L172Q) [17] has been analyzed thus far, which notably exhibited Lewy body (LB) pathology, suggesting a link between DJ-1 and α-synucleinopathy. The recent MDSGene Systematic Review, which focused on three autosomal recessive forms of PD (Parkin, PINK1, and DJ1), found that DJ-1 mutation carriers exhibit the highest percentage overall (57%) of non-motor symptoms [22]. This suggests that, in addition to being relevant to the motor symptoms in PD, DJ-1 function is also linked to other aspects of symptomology (anxiety, cognitive decline, psychotic symptoms). In agreement with these data, DJ-1 gene knockout mice, despite exhibiting a normal number of dopaminergic neurons in the substantia nigra, show subtle neurochemical and behavioral abnormalities, such as reduced dopamine overflow in the striatum, absence of long term depression, and hypoactivity in the open field [23,24].

**Figure 1:**
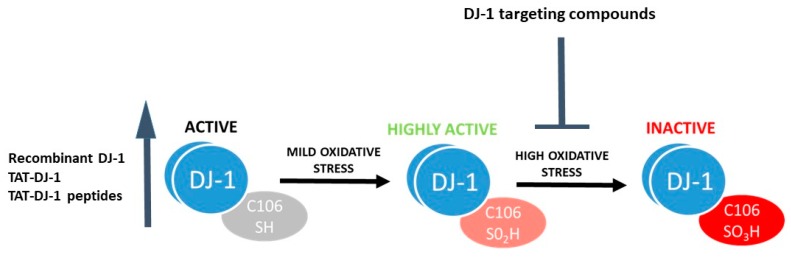
Targeting DJ-1 as therapeutic approach for PD. DJ-1 targeting compounds can either increase the activity of DJ-1 or stabilize the active form of the protein to obtain neuroprotection.

Strikingly, despite concerted efforts by the scientific community, it is still unclear how DJ-1 contributes to PD pathogenesis. Indeed, a plethora of different functions have been ascribed to DJ-1 (for detailed reviews see [25,26,27]), which complicates our understanding of how DJ-1 mutations specifically cause PD. In order to shed some light on DJ-1 biology, here we underline what we *do* know about DJ-1:

1. DJ-1 is highly expressed in cells with high energy demands, hence cells with higher levels of reactive oxygen species. Indeed, the majority of human cancers overexpress DJ-1 mRNA [28] and it is highly expressed in testicles, all of which are high-energy demand tissues characterized by a high glycolytic flux. Indeed, decreased expression of DJ-1 in sperm and epididymides correlates with male infertility [29,30]. DJ-1 protein levels also increase in human islets of Langerhans beta cells upon exposure to high glucose concentrations, indicating a protective role for DJ-1 in this cell type [31,32].
2. DJ-1 is involved in protection from oxidative stress, although the molecular mechanisms underlying these effects are not entirely clear. DJ-1 overexpression blocks oxidative damage, while oxidative stress-induced cell death increases in the absence of DJ-1 in cell culture and animal models [33,34,35,36,37,38]. Unfortunately, the molecular mechanisms underlying DJ-1 function remain elusive and a key outstanding question is how DJ-1 function is affected by its oxidative modification. Several review articles summarize what is currently known regarding DJ-1 protection from oxidative stress [39,40,41].
3. DJ-1 can sense oxidative stress. The protein structure of DJ-1 has been studied in detail [42,43,44], with a particular emphasis on the relevance of the conserved Cysteine (Cys) residue at position 106 for biological function. Cys106 is the preferential target for oxidative protein modification and is required for DJ-1 mediated protection from oxidative stress [45,46,47]. The reduced form of DJ-1 (DJ-1 Cys106-SH) can be oxidized to a sulfinic acid form (DJ-1 Cys106-SO_2_H) and a sulfonic acid form (DJ-1 Cys106-SO_3_H) in the presence of moderate or high oxidative stress paradigms (Figure 1). While the reduced and sulfinic DJ-1 forms are stable, the sulfonic form of DJ-1 is unstable and prone to aggregate formation [47,48,49].

Here we focus our attention on the clinical relevance of DJ-1 in neurodegeneration by overviewing: (1) The pathological features of DJ-1 in human brains, (2) the use of DJ-1 as a biomarker, and (3) the potential for DJ-1 as a target for therapeutic approaches.

## 2. DJ-1 in the Pathological Human Brain

Bandopadhyay and colleagues studied the distribution of DJ-1 in the human brain [50] and found that, while DJ-1 is not an essential component of LBs and Lewy neurites, it is highly expressed in astrocytes in the frontal cortex and substantia nigra of idiopathic PD brains, PD subjects with DJ-1 R98Q polymorphisms, and normal controls. Multiple DJ-1 isoforms were identified in control and PD brains, with the most alkaline pI isoform absent in a subset of the PD cases. This study was followed by the first analysis of DJ-1 mRNA levels in postmortem brain samples [51], which found decreased levels of DJ-1 mRNA and protein, as well as the presence of extra-oxidized DJ-1 isoforms in PD brains versus controls. In agreement with these data, acidic isoforms (pI 5.5 and 5.7) of the DJ-1 monomer were selectively accumulated in sporadic PD and Alzheimer’s disease (AD) brains compared with controls [52]. In cerebral ischemia, Mullet et al. [53] observed high DJ-1 protein expression in astrocytes in the infarcted area, both in the white and gray matter.

New possibilities for DJ-1 detection in the human brain have arisen from the development of specific antibodies against the oxidized form of DJ-1 [54,55]. Indeed, analyses of post-mortem brains from PD cases and controls using these antibodies have found oxidized DJ-1 immunoreactivity in cell bodies and neurites of neurons within the substantia nigra [54]. Interestingly, LBs in these samples are positive for oxidized DJ-1, with maximal staining in cases classified as LB Stage II and LB Stage III (LBs either without or with associated parkinsonism, respectively). Oxidized DJ-1 immunoreactivity was also present in astrocytes in the striatum and in neurons and glia in other central nervous system (CNS) regions related to movement regulation (red nucleus, inferior olivary nucleus). The main limitation of this study was the inability of the antibody to discriminate between the two different forms of oxidized DJ-1: Cys sulfinic acid (-SO_2_H, the predicted active form of DJ-1) and Cys sulfonic acid (-SO_3_H, the over-oxidized, inactive form of DJ-1). Although this currently remains an important target to achieve in order to completely understand the biological function of DJ-1, this study highlights that a close relationship exists between the oxidation state of DJ-1 and the progression of the disease. Further studies with larger human cohorts will be required to clarify these findings.

Another important aspect to be considered in the human brain is the localization of DJ-1 relative to the microtubule associated protein tau (MAPT). Abnormal tau hyperphosphorylation leads to the formation of neurofibrillary tangles in several tauopathies, such as AD, progressive supranuclear palsy (PSP), frontotemporal dementia and parkinsonism linked to chromosome 17 (FTDP-17), and corticobasal degeneration (CBD). DJ-1 co-localization with several forms of tau inclusions has been observed in tauopathies [56,57]. Strikingly, DJ-1 solubility was altered in association with its aggregation within these inclusions, while DJ-1 labelling was not detected within LBs in sporadic PD cases [57]. Differential expression of DJ-1 in tau pathological inclusions containing both 3R and 4R tau in neurodegenerative disorders has also been identified, thus suggesting a role for DJ-1 in the pathogenesis of tauopathies. A detailed analysis of DJ-1 and tau localization in brains from patients with mutated forms of DJ-1 would be of major help to clarify their interaction, as the only DJ-1-associated PD case that came to autopsy thus far [17] showed tau neurofibrillary tangles consistent with Braak neurofibrillary stage I/primary age-related tauopathy. Notably, DJ-1 is able to modulate toxicity/misfolding of two aggregation-prone proteins, α-synuclein (αSyn) [58,59] and mutant huntingtin (HTT) [60], likely due to a direct physical interaction between DJ-1 and the target protein. Furthermore, DJ-1 has been shown to act as a protein chaperone and inhibit microtubule associated protein 1B (MAP1B) aggregation both in vitro and in vivo [61]. Thus, it would not be surprising for DJ-1 to be present within tau inclusions due to this chaperone activity towards misfolded tau. Further studies will be needed to clarify this point.

## 3. DJ-1 as a Biomarker

The detection of biomarkers able to identify PD pathology in the early phases of the disease is a priority for the scientific community. In this regard, DJ-1 has been found to be constitutively present in the cerebrospinal fluid (CSF), and several groups have analyzed its potential as a biomarker for PD, with unclear and conflicting results (for a review see [62,63]). Notably, CSF DJ-1 levels were found to be significantly increased as a function of age, especially in controls, while the age dependence became weaker in patients with PD [64]. Moreover, CSF DJ-1 has also been studied as a useful parameter for a differential diagnosis between PD and other neurodegenerative diseases; however, no differences were found in DJ-1 CSF levels among PSP, dementia with LBs, or multiple system atrophy (MSA) individuals [65], while the combined detection of DJ-1 and total tau levels in CSF significantly improved the discrimination between MSA and PD.

A more convenient sample source for clinical application is blood; however, the extremely high concentration of DJ-1 in erythrocytes [66] makes the detection of plasma levels of DJ-1 challenging. A recent study explored DJ-1 levels in neurally derived exosomes from plasma [67], as a “window” into CNS changes [68]. This study showed that, despite there being no correlation with the progression of the disease, the ratio of neurally derived exosomal DJ-1 levels to total DJ-1 was increased in PD patients versus controls. Furthermore, a positive correlation was found between levels of DJ-1 and αSyn in plasma neural-derived exosomes, thus indicating a potential for exosomal DJ-1 as a PD biomarker. This is particularly important due to the high expression of DJ-1 in astrocytes and the role of astrocytes in cerebrovascular regulation and, therefore, the strong link with cerebral circulation. In 2006, Choi et al. [52] found the presence of oxidatively damaged DJ-1 in idiopathic PD and AD brains, and subsequent work detected a significant increase in oxidized DJ-1 in the erythrocytes of unmedicated PD patients versus medicated PD patients and healthy subjects, suggesting oxidized DJ-1 might be a useful biomarker for the detection and diagnosis of early stage PD [55]. A further step in this direction is the recent finding that oxidized DJ-1 in erythrocytes can be used as a biomarker for the differential diagnosis of PD [69]. Indeed, it is difficult to differentiate PD from other parkinsonian syndromes, such as PSP, MSA, and CBD, in the early stages of the disease. Notably, the levels of oxidized DJ-1 in erythrocytes were found to be higher in PD patients compared to patients with PSP or MSA, as well as the controls. A recent study of Korean PD individuals observed a two-fold increase in levels of oxidized DJ-1 in the urine compared with the controls [70]. Although promising, these studies need validation on larger human PD cohorts and healthy controls to fully ascertain the potential of oxidized DJ-1 as a general PD biomarker. In addition to assessing the status of oxidized DJ-1 in individuals, it may also be relevant to detect the reduced form of DJ-1 (-SH) in order to directly determine the ratios of reduced versus oxidized DJ-1 as this could be more informative and might have more power with regards to differential diagnosis. Indeed, in 2018 a chemical probe able to monitor DJ-1 in its reduced state in situ was identified [71], thus opening new perspectives for selective studies of DJ-1 as a biomarker.

## 4. DJ-1 as a Target for Therapeutic Approaches

Due to its ability to protect from oxidative stress, DJ-1 is an interesting target for therapeutic interventions. One approach, which is the most utilized thus far in different pathological models, is to increase DJ-1 levels to obtain neuroprotection when oxidative stress arises (Figure 1). The efficacy of recombinant wild type (WT) DJ-1 for protection of dopaminergic neurons has been demonstrated in several studies employing rat PD models [72,73,74]. However, all of these studies were performed with intranigral injection of recombinant DJ-1, far from clinical applicability. One step further was obtained by increasing DJ-1 intracellular expression levels through the addition of a TAT-fused recombinant protein, an approach that uses a TAT cell permeable peptide obtained from the transduction domain of the HIV-virus to cross the plasma membrane and deliver biological active proteins to all tissues [75]. This intervention resulted in reduced cell death after induction of oxidative stress in human neuroblastoma cells [58] and reduced 6-hydroxydopamine (6-OHDA) toxicity in vivo via intrastriatal administration [33]. In parallel, a peptide consisting of 13 amino acids of DJ-1 linked to 7 amino acids derived from TAT was found to decrease dopaminergic dysfunction and improve behavior in a 6-OHDA hemiparkinsonian mouse model, as well as attenuate 1-methyl-4-phenyl-1,2,3,6-tetrahydropyridine (MPTP) toxicity in vivo [76]. This peptide also provided neuroprotection in a mouse model of MSA [77]. Interestingly, TAT-DJ-1 peptides have been used with positive results in several other models of neurodegeneration where oxidative stress plays a major role in causing cell death, such as focal ischemic injury in mice [78] and ischemic damage in the rabbit spinal cord [79].

A second approach to target DJ-1 is the identification of drugs that inhibit excessive oxidation of Cys106. Overoxidation of DJ-1 with the consequent formation of DJ-1 sulfonic acid (DJ-1 Cys106-SO_3_) is considered an irreversible process leading to DJ-1 inactivation (Figure 1). Lin et al. [80] showed that the Cys106 sulfinate form (Cys-SO_2_^−^) results in thermal stabilization of both human DJ-1 and *Drosophila* DJ-1β and hypothesize that this stabilization may be a possible mechanism by which cysteine oxidation regulates DJ-1 function in vivo. Recent molecular dynamic simulations of DJ-1 oxidation states have found that despite the overall structure of DJ-1 being quite similar between states, significant differences are present that may impact upon its stability and function [49]. Thus, one promising strategy for obtaining DJ-1 mediated protection may be to prevent its overoxidation. With this hypothesis in mind, Ariga and his group carried out virtual screening of compounds using the crystal structure of the reduced and SO_2_H-oxidized Cys106 region of DJ-1 and identified several brain penetrant molecules able to interact with the Cys106 region of DJ-1 that help maintain its active form [81]. Interestingly, these DJ-1 interactors prevented dopaminergic neuronal death, restored normal locomotor function in rodent models of PD, and conferred protection in rat cerebral ischemia [82,83,84,85]. The most promising compound identified, Compound-23, was found to inhibit MPTP-induced locomotor deficits and cell death in the substantia nigra and striatum, as well as rescue dopamine content in an MPTP-treated mouse model of PD [86]. Another compound, Compound-B, has subsequently been tested in a transgenic mouse model of AD and found to improve spatial learning, memory, and amyloid-β clearance, thus suggesting for the first time that DJ-1 compounds may have applications for AD treatment [87]. The above studies employed toxin models of PD, characterized by oxidative stress and cell death in dopaminergic neuronal cells. Despite oxidative stress being an important player in PD, acute toxin models recapitulate only a subset of normal disease etiology associated with human PD. Thus, it would be relevant to the field to test DJ-1-targeted strategies in gene-based models that may better represent clinical PD. Notably, increased expression levels of A53T αSyn negatively correlate with DJ-1 expression levels in A53T αSyn mice exposed to subtoxic doses of MPTP [88], indicating that an interplay between DJ-1 and αSyn exists and suggesting that targeting DJ-1 as described above could be relevant.

## 5. Conclusions and Future Directions

The findings described above highlight the relevance of DJ-1 as a promising biomarker and therapeutic target for PD, as well as a broader range of neurodegenerative diseases. Upcoming studies will need to further characterize DJ-1 within the CNS in pathological conditions, with a specific focus on its aggregation and oxidation state. In this regard, a key aspect for future work will be the development of antibodies for the detection of DJ-1 specific oxidation and aggregation states. Such studies using human cohorts are currently missing and would represent an important step in unravelling the potential of DJ-1 as a PD biomarker. Due to its role in protection from oxidative stress, DJ-1 also represents an ideal target for therapeutic intervention and further work will clarify the feasibility of utilizing DJ-1 targeting compounds that are able to either stabilize the active DJ-1 form or increase DJ-1 activity to obtain neuroprotection.

## Figures and Tables

**Table 1:** Overview of pathogenic DJ-1 mutations in Parkinson’s disease (PD).

*PARK7* Mutation	Location	References
g.159C > G + IVS4 + 3insA: Compound het	5′ UTR; Intron 4	[4]
Ex1-5del (g.4443-18524del): Hom	Exons 1–5	[1]
Leu10Pro: Hom	Exon 2	[5]
Thr19Lysfs*5 + IVS6-1G→C: Compound het	Exon 2; Intron 6	[6]
Asp24Metfs*3: Hom	Exon 2	[7]
Met26Ile: Hom	Exon 2	[2]
Ile31Aspfs*2: Hom	Exon 2	[8]
g.11032A>G: Hom	Intron 2	[9]
Gln45*: Hom	Exon 3	[10]
Glu64Asp: Hom	Exon 3	[11]
Ex5del: Hom (breakpoints not detailed)	Exon 5	[12]
Ile105Phe: Hom	Exon 5	[13]
Ala107Pro: Hom	Exon 5	[9]
g.16677 A > C: Hom	Intron 5	[14]
Thr154Lys: Hom	Exon 7	[15]
Pro158del: Hom	Exon 7	[16]
Glu163Lys: Hom	Exon 7	[3]
Leu166Pro: Hom	Exon 7	[1]
Leu172Gln: Hom	Exon 7	[17]
